# On the Treatment of Tuburcular Hemoptysis

**Published:** 1862-09

**Authors:** William Brinton

**Affiliations:** Physician to St. Thomas’s Hospital


### ON THE TREATMENT OF TUBERCULAR HEMOPTYSIS.

By Dr. WILLIAM BRINTON, Physician to St. Thomas's Hospital.

Of course, pulmonary hemorrhage has the usual long list of specifics, each of which, in the eyes of its partizans is so efficacious that one can hardly avoid wondering anybody should be so foolish as to die of hemoptysis at all. Alum, turpentine, mercury, the mineral acids, acetate of lead, sulphate of copper, tanic acid and a host of analogous astringents, ipecacuanha, tartar emetic, the external application of ice, figure amongst the favorite remedies of this kind. The variations of diet have been scarcely less striking than those of drugs; the patient who would have been nearly starved twenty years ago to favor coagulation of the blood in the ruptured vessels, being now sometimes crammed with food, and drenched with stimulants, at frequent intervals, in order to "keep up his strength."

Warned by such discrepancies, I think we are justified in regarding as inconclusive what are often termed the "teachings of experience"—namely, the mere events which follow the adoption of this or that drug or dietary in the treatment of the malady. Here, as elsewhere in the study of therapeutics it is our first duty to check the uncertainty or bias which must sometimes beset the conclusions of the most judicial intellect, by a study of the nature and history of the disease, and by the physiological action of the remedy. What *is* hemoptysis, on the one hand? What does the particular drug do to the healthy body, on the other?

Unless we can at least partially answer the first question, it is difficult to see how we can conclude any remedy really useful. Unless you can answer the second, and point to at least an analogy between the effects of the drug in health and in disease, it is nothing short of a very complete and large account of the natural course of the malady that would enable us to accept any recovery as a cure. True it is that quinine cures most forms of ague, and mercury some forms of syphilis, by agencies which are quite inexplicable from the mere physiological effects of either drug respectively. But here our knowledge of the course of the two maladies, unmodified by their respective specifics, proves the reality of the cure. It is not so much a multiplication of coincidences, as an asservation of cause and effect, which is thus afforded us.

Looking back to the variations of hemoptysis already alluded to, and eliminating the casual bleedings of pleurisy, and the more essential, but dilute, hemorrhages of pneumonia, you will remember that we noticed the hemoptysis of pulmonary tubercle as presenting itself in three forms of very different frequency and gravity. The afflux, or active congestion, of the first stage of tubercular deposit, and the obstruction and erosion in the second stage, affect chiefly the smaller vessels of the diseased organ. The large ulceration of the third stage occasionally lays open a bloodvessel of considerable magnitude. The first two are of frequent occurrence; indeed, the second is, in greater or less extent, almost a necessary incident of the malady. The latter is fortunately rare.

Now, of these two commoner varieties of tubercular hemoptysis, it may be unhesitatingly affirmed, that in a very large percentage of their cases the bleeding would cease spontaneously. Indeed, inquiry amongst the poorer classes, who form the outpatients of our hospitals, shows that hemorrhage of this kind commonly ceases in a day or two: not only without medical advice, but often in defiance of the want of that perfect rest and unstimulating diet which, apart from drugs, constitute the best means of favoring its arrest. And I need hardly say that, in such a process, Nature is only stanching an internal bleeding by the same means which she ordinarily employs to arrest an external one; means which, though in the case of these pulmonary vessels they have to fight against additional obstacles in the shape of the alternate suction and compression of breathing, and the agitation of cardiac movement, are yet in the main sufficient.

Hence, to assert that these frequent and scanty hemorrhages generally cease after the administration of any of the above drugs, is to state a fact which, the moment it is made to sustain an assertion of their specific operancy as stiptics, may be challenged as an instance of the fallacy "*post hoc, ergo propter hoc;*" for the fact is utterly insufficient to sustain any such conclusion. The bleeding may have ceased spontaneously; a recovery which, supposing the instincts of the patient have been allowed full play in the matter of rest and diet, is even additionally explicable. Strictly speaking, the mere coincidence scarcely entitles us to affirm more of stiptic power in the drug than if the bloodvessels concerned had been those of a cut finger.

In all cases of hemoptysis, the first indication of treatment is suggested by the pathology of these, and indeed most other, morbid hemorrhages. That the bleeding is generally preceded and accompanied by great congestion of the bloodvessels nearest the lesion is a fact about which there can be no doubt whatever. And that the removal of this condition can often be effected by smart aperients is another fact which may be as unhesitatingly asserted. Nowhere are these statements better verified than in the digestive canal; where, for example, you may sometimes see in the submucous areolar tissue that the nodule of deposit which has excited a copious hemorrhage, is surrounded by an inch or two of deep velvety congestion, with a well-defined margin towards the neighboring healthy structures; and where the virtues of drastic purgatives in melæna constitute an experience as old as the days of Hippocrates. And all the purgatives which one may give with this purpose, the old-fashioned dose of blue-pill or calomel, followed by a black draught, seems really the quickest and most efficacious. Under all ordinary circumstances its administration, and, if needful, repetition, should be a prominent feature of the treatment.

Then as to the drugs for checking hemorrhage, by their astringency or otherwise. There seems to be a kind of notion in the minds of some therapeutical writers, that such drugs as turpentine, alum, gallic acid, acetate of lead, sesquichloride of iron, etc., etc., when taken into the stomach, are received into the blood in quantities sufficient to communicate to this fluid those styptic properties which they undoubtedly possess when applied to an external wound, or to a bleeding stomach, or perhaps (through the urine) to a bleeding bladder. But I know of nothing to confirm such a notion. And I think that those who would look the facts of therapeutics sturdily in the face (which, not wishing to die a homœopath, I intend to do), would scarcely think twice about the matter. At any rate experiment would soon afford some analogical answer. Stir up 10 grains of gallic acid, or 10 minims of tincture of sesquichloride of iron, in 10 or 20 pints of blood, and I think the resulting solution would be a styptic of a somewhat Hahnemannic degree of diluteness; not nearly so potent, one may anticipate, as the compound tincture of benzoin which, forty years ago, was the favorite application of some old-fashioned country surgeons to stanch the bleeding of a superficial wound, and which, in its turn, modern surgeons have practically superseded by a little patience and cold water.

Of other remedies for hemoptysis one may speak more respectfully, though with some qualifications. Ice, for example, seems occasionally of service. But the internal congestion, invariably produced by great external cold, suggests that, save where the source of the hemorrhage is near enough to the surface to be directly affected by it, its benefits may be more than questionable. And of ipecacuanha, which many excellent authorities think highly of, in small and frequent doses, (a grain every ten or fifteen minutes,) I can only say that I have known its use followed by great exhaustion and nausea, and a very protracted convalescence, but, (what is more to the purpose,) that over and over again I have known it utterly fail in any degree to moderate such a hemorrhage.

In short, all else that I have to say of such remedies might almost be summed up in the statement, that it is in the terrible hemorrhage of larger eroded vessels, and therefore of what is often advanced phthisis, that we have the most searching test of the value of any drug of this kind. Presuming we have such a means, we must very frequently use it; for it is often impossible to tell beforehand whether a slight oozing is not the commencement of a more considerable hemorrhage, such as (directly or indirectly) endangers life. And I believe that we have such a remedy, for the majority of patients, in *digitalis*.

The grounds for the strong personal conviction I find chiefly in the fact, that I have repeatedly seen profuse and alarming hemoptysis, which had resisted many other means of treatment, including a most judicious diet, and all the other drugs above enumerated, yield to this active remedy, given so as to produce its well known specific effects on the pulse. And not only so, but, in several cases, the interruption of the drug seems to have permitted the return of the hemorrhage, to be again arrested by the resumption of the digitalis. Our patient in bed No. 9, of Jacob's ward, so far illustrates this point as that, thrice over, the gradual disuse of the digitalis, at intervals of two and three weeks from the previous cessation of the hemoptysis under the use of the drug, has been followed by fresh attacks. And the very same physiological effects which confirm the efficacy of digitalis may suggest the details and qualifications of its use. Pray remember that it is too much of an edged tool to be carelessly handled; and hence that, if you cannot or will not take the trouble to watch over its administration, you had best not give it at all. From thirty to ninety minims of the officinal tincture daily, in four or six doses, is as much as is generally requisite; and, (as has been pointed out by Sir Henry Holland, in that chapter of his classical work devoted to the consideration of this drug,) it is often not the robust, strong man who bears or demands the largest dose. Keep the patient quiet, and in the recumbent posture—an attitude for which the drug is a double argument in this malady. Judge, by repeated enquiry, how far, or how quickly, the digitalis is acting on the pulse; and, as a rule, reduce the dose as soon as the pulse has dropped to the slow rate, (30 to 50 or 60 per minute,) which is the usual result of its action both in health and in disease. Lastly, for those who like polypharmacy, the drug combines well enough with dilute sulphuric acid during the attack, and with sesquichloride of iron during the convalescence, without substantial detriment to its own much greater virtues.—*Lancet, Nov.* 30, 1861, *p.* 516; *Braithwaite's Retrospect.*

